# PLGA nanoparticle encapsulation reduces toxicity while retaining the therapeutic efficacy of EtNBS-PDT *in vitro*

**DOI:** 10.1038/srep33234

**Published:** 2016-09-30

**Authors:** Hsin-I Hung, Oliver J. Klein, Sam W. Peterson, Sarah R. Rokosh, Sam Osseiran, Nicholas H. Nowell, Conor L. Evans

**Affiliations:** 1 Wellman Center for Photomedicine, Harvard Medical School, Massachusetts General Hospital, 149 13th Street, Charlestown, Massachusetts 02129, United States; 2 Harvard-MIT Division of Health Sciences and Technology, 77 Massachusetts Avenue E25-519, Cambridge, Massachusetts 02139, United States

## Abstract

Photodynamic therapy regimens, which use light-activated molecules known as photosensitizers, are highly selective against many malignancies and can bypass certain challenging therapeutic resistance mechanisms. Photosensitizers such as the small cationic molecule EtNBS (5-ethylamino-9-diethyl-aminobenzo[a]phenothiazinium chloride) have proven potent against cancer cells that reside within acidic and hypoxic tumour microenvironments. At higher doses, however, these photosensitizers induce “dark toxicity” through light-independent mechanisms. In this study, we evaluated the use of nanoparticle encapsulation to overcome this limitation. Interestingly, encapsulation of the compound within poly(lactic-co-glycolic acid) (PLGA) nanoparticles (PLGA-EtNBS) was found to significantly reduce EtNBS dark toxicity while completely retaining the molecule’s cytotoxicity in both normoxic and hypoxic conditions. This dual effect can be attributed to the mechanism of release: EtNBS remains encapsulated until external light irradiation, which stimulates an oxygen-independent, radical-mediated process that degrades the PLGA nanoparticles and releases the molecule. As these PLGA-encapsulated EtNBS nanoparticles are capable of penetrating deeply into the hypoxic and acidic cores of 3D spheroid cultures, they may enable the safe and efficacious treatment of otherwise unresponsive tumour regions.

Ovarian cancer (OvCa) is the leading cause of gynaecologic cancer death and is the 5^th^ most common cause of cancer death in women in the United States^1^. The most common type, epithelial ovarian cancer (EOC), accounts for 90% of ovarian cancer cases^1^. Due to a lack of early detection methods and non-specific symptoms, over 75% of OvCa patients are diagnosed with metastatic, advanced-stage disease. The current standard treatment for OvCa is cytoreductive surgical debulking followed by platinum- and taxane-based combination chemotherapy. Although 80–90% of EOC patients are initially responsive, nearly 70% of these patients will develop resistance to chemotherapy over time^2^. This evolution of therapeutic resistance in EOC results in a poor quality of life for patients and a dismal overall five-year survival rate of 30%^1^,^2^,^3^. As such, significant efforts have focused on combating treatment resistance early in the therapeutic process.

Microenvironmental factors, including hypoxia and acidosis, are known to play a role in treatment resistance. Hypoxia is recognized as a major obstacle in cancer therapy due to the resulting upregulation of a host of cellular survival mechanisms, including the HIF1 cellular survival pathway. An acidic tumour environment has similar cellular protective effects, partly through hindering drug uptake^4^,^5^,^6^,^7^,^8^,^9^,^10^. Of particular importance to OvCa, numerous reports have suggested that tumour initiating cells (TICs), also known as cancer stem cells (CSCs), and their associated tumour microenvironment play a significant role in the development of therapeutic resistance and treatment failure^2^,^3^,^4^,^5^,^11^. TICs are thought to inhabit protective hypoxic niches^4^,^12^. and TIC-associated tumour relapse and post-therapy metastasis is postulated as a major cause of patient death. Targeted cellular killing of TICs and the disruption of their associated microenvironment may therefore be an effective approach to eliminating resistant OvCa early in the treatment process.

Photodynamic therapy (PDT) is a light-activated therapeutic modality that makes use of molecules known as photosensitizers. When exposed to specific wavelengths of light, photosensitizers generate reactive radical species that can cause apoptosis or necrosis^13^. This approach reduces systemic toxicity when compared to chemotherapy, as photosensitizers are activated only when exposed to light. Direct targeting of cellular organelles, a general lack of nuclear or DNA damage, and the preferential uptake of photosensitizers into tumours give PDT many advantages over traditional chemotherapy and radiotherapy^13^,^14^. As a result, PDT has great potential to overcome adaptive therapeutic resistance^15^,^16^,^17^. In both basic and clinical studies, PDT was found to not contribute to the rise of therapeutically resistant cell populations, even if used repeatedly^14^. A number of photosensitizers are available for PDT, with the FDA-approved, porphyrin-based Photofrin having been used clinically for over three decades for a range of malignancies^18^,^19^. PDT has found numerous clinical applications, including the therapy of mesothelioma^20^, prostate cancer^21^, pancreatic cancer^22^, head and neck cancer^23^, and glioblastoma^24^. PDT treatments against metastatic OvCa have shown promise in pre-clinical experiments^25^ and are currently undergoing testing in a Phase I clinical trial. For these reasons, PDT appears well suited for the treatment of OvCa.

There are two types of radical photochemistry that can occur during PDT^26^. The most well-known process is oxygen-dependent and proceeds through energy exchange between a photosensitizer’s excited triplet state and molecular oxygen. This “Type II” photochemical mechanism causes the efficient generation of singlet oxygen molecules that then impart cellular cytotoxicity. In the second mechanism, excited photosensitizers^27^ directly react with their surroundings via their excited states. This so-called “Type I” mechanism proceeds via electron transfer to water, proteins, and lipids to create reactive species including the hydroxyl radical (∙OH) and hydrogen peroxide (H_2_O_2_). Though potentiated by oxygen, this second mechanism can operate independently of oxygen, making it useful against cells in hypoxic microenvironments.

PDT agents currently used in the clinic operate primarily through the oxygen-dependent Type II mechanism, meaning that these agents are largely ineffective in hypoxic environments. Recently, we and others found that a lysosome-targeted photosensitizer, EtNBS, specifically accumulated into otherwise therapeutically unresponsive acidic and hypoxic tumour microenvironments and was capable of killing cells within these problematic regions^28^,^29^. Importantly, this cationic molecule was found to trigger cell killing through a combination of Type I and II mechanisms and was found to retain its therapeutic efficacy even under severely hypoxic conditions^28^,^29^,^30^,^31^. As hypoxic microenvironments are traditionally difficult to treat with chemotherapy and radiation therapy, combination therapeutic regimens with EtNBS may offer new options for patients^29^. The molecule’s localization to acidic environments and its potency under hypoxic conditions also make EtNBS a potent agent for the treatment of unresponsive tumour regions, as well as a potential therapeutic for killing TICs in their niche. The current drawback to EtNBS is that it demonstrates dark toxicity – toxicity in the absence of light activation – at high micromolar concentrations^32^. As EtNBS can accumulate within cells and tissues at such concentrations, approaches are needed that can lessen or ameliorate dark toxicity.

Nanoparticle encapsulation provides one solution to minimize the potential dark toxicity of EtNBS. Poly(lactic-co-glycolic acid) (PLGA) is a biodegradable and biocompatible material that has been FDA-approved for the safe delivery of drugs^33^. PLGA can be readily formulated into nanoparticles for the encapsulation of numerous hydrophilic compounds, including chemotherapeutics^34^, analgesics^35^, and anti-inflammatories^36^. Moreover, the conjugation of PLGA to various drugs has been very well documented^33^,^37^,^38^. In this study, we sought to encapsulate EtNBS within PLGA nanoparticles to reduce the dark toxicity associated with its free delivery. Although it was assumed that this would reduce the overall efficacy of EtNBS-PDT, we saw no such reduction *in vitro* due to a photodynamic release mechanism that liberates EtNBS from the nanoparticle in a light-dose dependent fashion. This retained efficacy, along with the ability to co-encapsulate other therapeutic agents, makes PLGA-EtNBS constructs promising agents for future pre-clinical studies.

## Results

### PLGA-encapsulated EtNBS nanoparticles show reduced dark toxicity compared to free EtNBS

**Figure 1:**
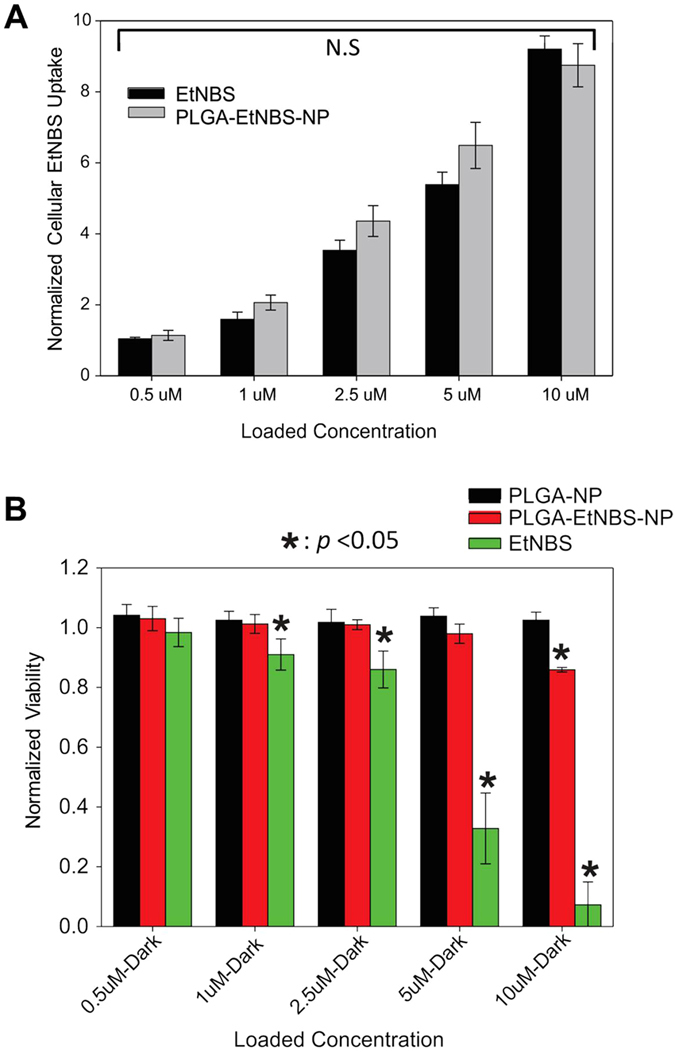
Comparison of uptake and dark toxicity of free EtNBS and PLGA-EtNBS nanoparticles. (**A**) Cellular uptake of free EtNBS and PLGA-EtNBS at equivalent cellular incubation concentrations. (**B**) Dark toxicity caused by PLGA vehicle controls (PA), free EtNBS, and PLGA-EtNBS nanoparticles (PE) at equivalent incubation concentrations/equivalent volumes. Viability was determined via the MTT cell viability assay.

The delivery and dark toxicity of EtNBS physically encapsulated in PLGA nanoparticles (PE) were first compared to control PLGA nanoparticles alone (PA) and free EtNBS. Characteristics of the nanoparticles measured by dynamic light scattering (DLS) can be found in Table 1. The size of PE nanoparticles were found to be approximately 170 nm, with zeta potentials ranging from −34 to −41 mV, indicating good particle stability in solution. OVCAR5 cells were loaded with 0.5 μM, 1 μM, 2.5 μM, 5 μM and 10 μM of either free EtNBS or the equivalent EtNBS concentration physically encapsulated in PLGA (PE) in suspension for 1.5 h. Collected cell pellets were processed as described in the Methods section to release EtNBS from the delivered PLGA nanoparticles. The resulting solutions were measured with a fluorometer to assess the degree of EtNBS cellular uptake (Fig. 1A). All readings were normalized to that of cells exposed to free EtNBS at 0.5 μM. For any given EtNBS dose, no statistically significant difference in uptake was observed between free and physically encapsulated EtNBS. Additional uptake experiments were carried out with 45 min and 3 h incubation periods (data not shown). These experiments revealed that the uptake profile of PLGA-EtNBS is similar to that of free EtNBS^20^,^21^, plateauing between 45 and 90 min. For this reason, 1.5 h of incubation time was used throughout all experiments with EtNBS in monolayer OVCAR5 cultures.

Next, to explore the effect of nanoparticle encapsulation on EtNBS dark toxicity, OVCAR5 cells were loaded with either PE nanoparticles or free EtNBS at doses from 0.5 μM to 10 μM for 1.5 h. Empty PLGA nanoparticle controls (PA), otherwise identical to the EtNBS-loaded PLGA nanoparticles (PE), were also loaded into OVCAR5 cells at volumes equivalent to those of the corresponding PE nanoparticles. Measured via the MTT assay, the PA control did not cause any dark toxicity compared to untreated cell controls (Fig. 1B). Free EtNBS began to show significant dark toxicity starting at 1 μM (p < 0.05). By 10 μM, free EtNBS caused nearly complete toxicity without light activation (Fig. 1B). In contrast, the PE nanoparticles demonstrated reduced dark toxicity, with protective effects observed even at 10 μM equivalent doses (Fig. 1B). These results confirm the ability of PLGA nanoparticles to deliver EtNBS with reduced dark toxicity, even at doses that would otherwise be completely cytotoxic.

### PLGA-encapsulated EtNBS nanoparticles maintain PDT efficacy

While PLGA nanoparticle encapsulation was able to reduce the dark toxicity of EtNBS, it was not clear if the EtNBS contained within would still be available for PDT. To determine the therapeutic efficacy of the PE nanoparticles, we next evaluated their PDT efficacy as compared to free EtNBS. PE and free EtNBS were incubated with cells at doses ranging from 0.5 μM to 2.5 μM and treated with 1 J/cm^2^, 5 J/cm^2^, and 10 J/cm^2^ light doses at 660 nm. As free EtNBS concentrations of 5 μM and 10 μM caused 70% and 90% dark toxicity, respectively, comparisons between PE and free EtNBS were not made at these doses.

**Figure 2:**
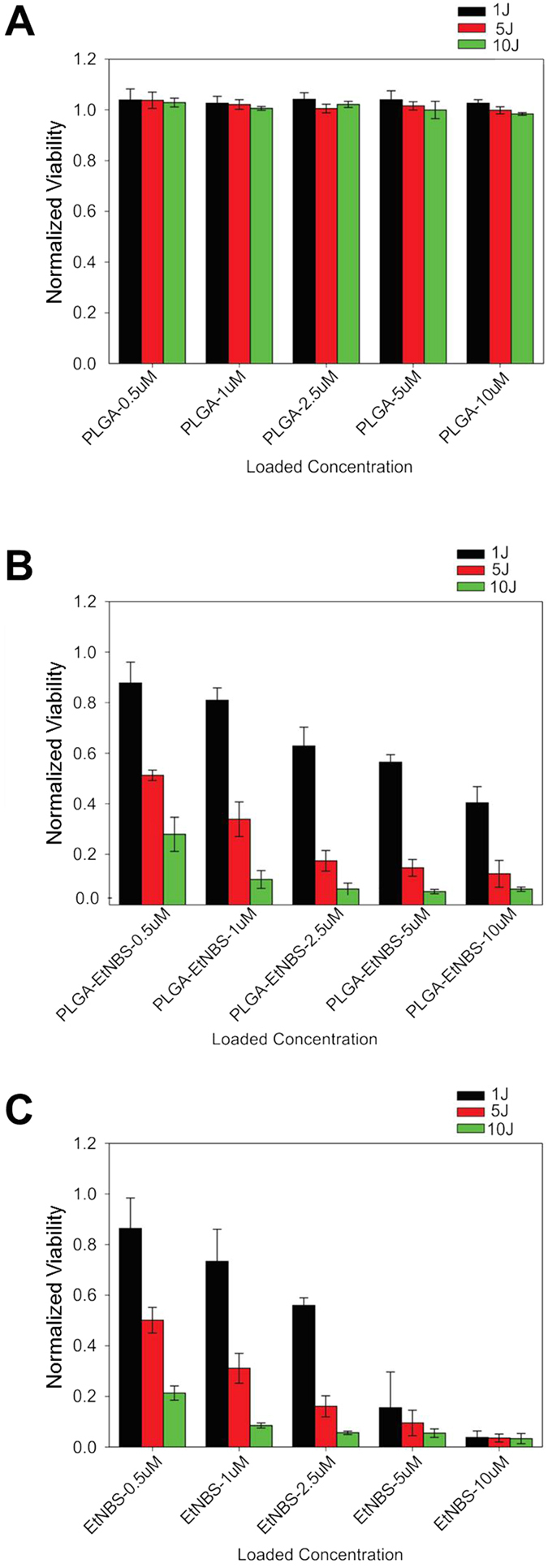
PDT-induced death of OVCAR5 cells in monolayer culture. (**A**) Viability of cells to PLGA vehicle only controls, showing no observed toxicity at any light dose. (**B**) Viability of cells following PLGA-EtNBS PDT across a range of concentrations and light doses. (**C**) Viability of cells following EtNBS-PDT across a range of concentrations and light doses. Viability was determined via the MTT cell viability assay.

PA nanoparticles, administered as vehicle-only controls, showed zero cellular killing effect across all light doses and compound concentrations, as expected (Fig. 2A). For the PE nanoparticles, PDT efficacy positively correlated with both the light dose and delivered photosensitizer dose (Fig. 2B). The treatment efficacy of the PE nanoparticles was found to correspond well to that of free EtNBS at both 500 nM and 1 μM doses, concentrations where EtNBS dark toxicity was minimal or non-existent. Despite the free EtNBS dark toxicity observed at 2.5 μM (Fig. 1B), the cellular viability of PE and free EtNBS (Fig. 2B,C) was still found to be similar, differing only by the degree of dark toxicity observed in Fig. 1B. This similarity in PDT efficacy between PE and free EtNBS delivery demonstrates that even though encapsulation within the PLGA nanoparticle protects cells from the cytotoxic effects of high levels of EtNBS, the photosensitizer contained inside retains its therapeutic efficacy. It is hypothesized that this occurs through two sequential processes: (1) PLGA nanoparticles are endocytosed within cells and (2) upon excitation, EtNBS is released from the nanoparticles intracellularly.

### PLGA-encapsulated EtNBS nanoparticles likely undergo endocytosis into lysosomes

PLGA nanoparticles have been shown to deliver their cargo via several mechanisms, including “kiss-and-run” and endocytotic mechanisms^39^. Though there is a possibility that EtNBS is delivered to the cells via a kiss-and-run mechanism, a process through which compounds within PLGA nanoparticles are transferred into endosomes at the cell surface, it is unlikely to be the case here, given the similarity between PE and free EtNBS PDT efficacy. To better determine which mechanism (endocytosis vs. kiss-and-run) was contributing to EtNBS nanoparticle uptake, a chemically conjugated (CC) form of the nanoparticle was designed, where EtNBS was synthesized with an amine terminal group that was covalently linked to PLGA. If delivery of PLGA-EtNBS was mediated by a kiss-and-run mechanism, we would not expect to see the uptake of the CC nanoparticles.

**Figure 3:**
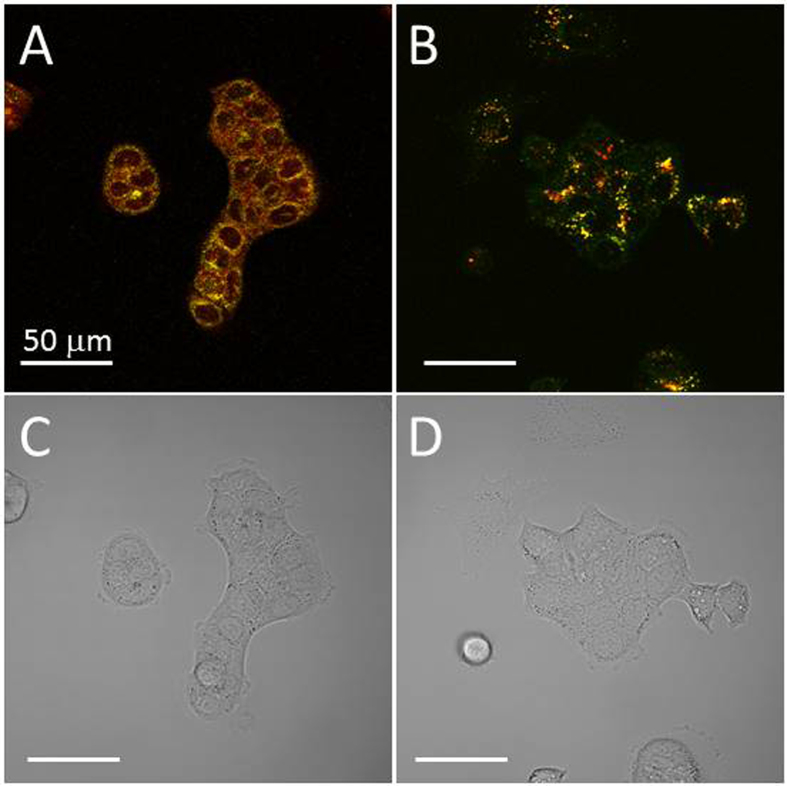
Physically encapsulated (PE) and chemical conjugated (CC) PLGA-EtNBS nanoparticles are both endocytosed into lysosomes. (**A**) Uptake of PE nanoparticles into OVCAR5 cells. EtNBS fluorescence is in red, while Lysotracker Green is displayed in green. (**B**) Uptake of CC nanoparticles, with EtNBS in red and Lysotracker in green. Note that in the CC nanoparticle case, the EtNBS signal is solely localized in lysosomes, while the PE nanoparticles demonstrate more diffuse EtNBS signal. This is thought to arise from minor leakage during the incubation period of EtNBS from the PE nanoparticles into the endoplasmic reticulum. (**C,D**) Trans-illumination images of (**A,B**), respectively.

CC nanoparticles, formed through nanoprecipitation, were co-delivered to cells along with Lysotracker. EtNBS fluorescence was found to be co-localized with the Lysotracker signal, demonstrating that CC nanoparticles are endocytosed into cells and localize primarily within lysosomes (Fig. 3). PE nanoparticles, when delivered to cells under similar conditions, also demonstrated primarily lysosomal localization. Furthermore, the uptake of CC nanoparticles across all administration doses, from 50 nM to 5 μM, and incubation periods, from 45 min to 90 min, was found to be very similar to the uptake of both free EtNBS and PE nanoparticles, with EtNBS fluorescence plateauing between 45 min and 1.5 h (data not shown). As both CC and PE nanoparticles were found to have similar sizes and zeta potentials (Table 1), endocytosis is likely a major mechanism of PLGA-EtNBS nanoparticle cellular uptake.

### PLGA-encapsulated EtNBS nanoparticles release EtNBS by a light-dependent mechanism

**Figure 4:**
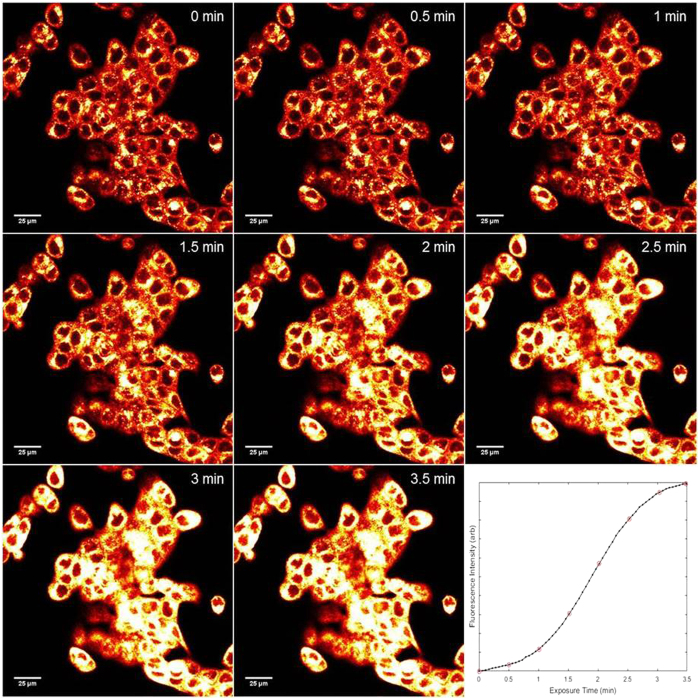
Photobrightening of PLGA-EtNBS nanoparticles over time. Cells were continuously observed over 3.5 min on a confocal microscope using 1.4 mW of focused 635 nm excitation raster-scanned across the field of view to visualize EtNBS via its 670 nm-centred fluorescence (via a 60X, 1.20NA objective, corresponding to a field of view size of 212 × 212 μm^2^, 512 × 512 pixels, a pixel dwell time of 4 μs/pixel, and 3x line averaging, resulting in an acquisition time of 3.3 seconds per frame). Initially, EtNBS fluorescence is weak and localized to lysosomes. As the 635 nm light is continuously scanned, the fluorescence intensity increases and eventually plateaus, as shown in the inset depicting the increase in fluorescence intensity across the field of view.

While imaging the localization of EtNBS nanoparticles in cells, it was observed that instead of photobleaching under constant illumination, cells incubated with the PE form photobrighten over time (Fig. 4). Moreover, the greater the light irradiance, the more rapidly this photobrightening occurred. Throughout the photobrightening process, the localization pattern of EtNBS fluorescence was observed to change from its original punctate appearance to a largely diffuse pattern spanning entire cells (see Supplementary Video S1). This change in localization pattern closely matches the spread of free EtNBS observed within cells following irradiation observed previously^29^,^40^,^41^. EtNBS-PDT has been shown to disrupt lysosomal integrity, causing EtNBS to diffuse throughout the cell body. The strong similarity between both free and nanoparticle-delivered EtNBS redistribution during PDT suggests that the photosensitizer released from lysosomes in these experiments is no longer nanoparticle-bound.

Such a photochemical mechanism, known as photodynamic release (PDR), has been observed with other photoactive compounds^42^. EtNBS molecules are well known to undergo ground state quenching when placed into close proximity^43^; this is why PE and CC nanoparticles must first be broken down and dissolved prior to spectrophotometric or fluorescence quantification. As a photosensitizer, EtNBS interacts with its environment to produce radical species that mediate its cytotoxic potential. The data suggest that this photochemical process can create radical species that react with the nanoparticles’ PLGA matrix, generating pores and channels through which EtNBS is released. Although EtNBS within particles is in a quenched state, this quenching is not complete^43^, enabling a fraction of molecules to absorb photons and react.

### PLGA-encapsulated EtNBS nanoparticles remain potent under hypoxic conditions

**Figure 5:**
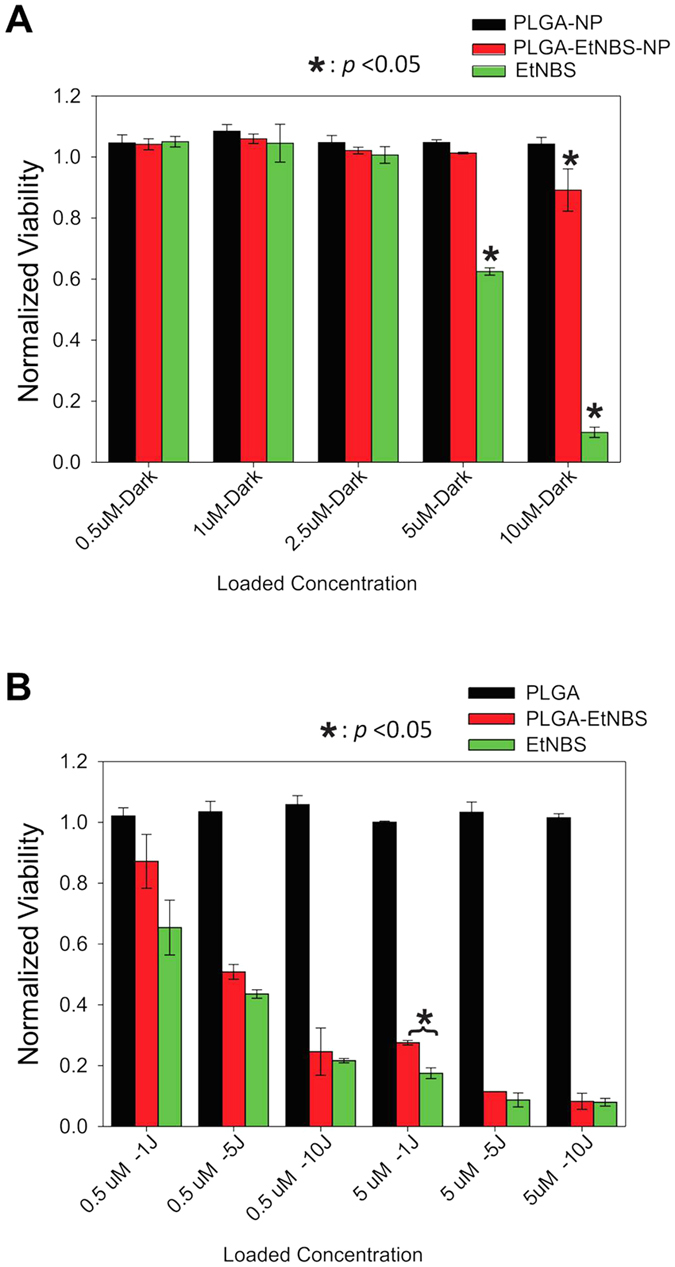
PDT of EtNBS and PLGA-EtNBS nanoparticles at equivalent loading concentrations under hypoxic conditions. (**A**) Viability of cells following incubation of each agent under hypoxia without the application of light. (**B**) Viability of cells under hypoxia following PDT at 0.5 μM and 5 μM equivalent EtNBS doses at different light doses. Note that PLGA-EtNBS nanoparticles have similar efficacy to free EtNBS even under hypoxic conditions. Viability was determined via the MTT cell viability assay.

One of the attractive prospects for the use of EtNBS in the treatment of cancer is its ability to impart cytotoxicity in severely hypoxic environments, leading to the killing of otherwise therapeutically unresponsive cell populations. Prior reports of PDR made use of primarily type II photosensitizers that act via the generation of singlet oxygen, which makes such particles ineffective at low oxygen partial pressures. As EtNBS can react via both oxygen-dependent and oxygen-independent pathways, it was important to determine if PE nanoparticles were still effective under hypoxic conditions. The effects of PDT under hypoxic conditions are shown in Fig. 5. For the PA control and PE nanoparticle treatment groups, cells experience no significant difference in dark toxicity between normoxic and hypoxic conditions (Fig. 5A). Interestingly, free EtNBS still was found to cause dark toxicity in a dose-dependent manner, but was found to be less toxic in hypoxic environments than in normoxic conditions, likely a result of reduced reactive oxygen species generation. At a concentration of 5 μM and under hypoxic conditions, free EtNBS was observed to cause significantly less dark toxicity than under normoxic environments at the same concentration (p < 0.05) (Fig. 5A).

To assess the PDT efficacy of PE nanoparticles under severe hypoxia, OVCAR5 cells were pre-treated for 6 h in the hypoxia chamber. As expected, cells in the PA control group were not found to experience any cytotoxicity (Fig. 5B). Importantly, both PE and free EtNBS showed similar levels of cytotoxicity under hypoxia, demonstrating that the PE nanoparticles were capable of releasing their EtNBS cargo even under hypoxic conditions (Fig. 5B). Collectively, the results shown in Fig. 5 demonstrate that under hypoxia, the PLGA-EtNBS nanoparticles mitigated EtNBS dark toxicity while still maintaining PDT efficacy similar to that of EtNBS. Additionally, the effectiveness of the PLGA-EtNBS nanoparticle was unchanged under hypoxic conditions. The effective killing of cells in hypoxic environments indicates that photodynamic release of EtNBS can still be triggered by a Type I mechanism in low oxygen conditions.

### PLGA-encapsulated EtNBS nanoparticles demonstrate uptake throughout three-dimensional tumour culture models

**Figure 6:**
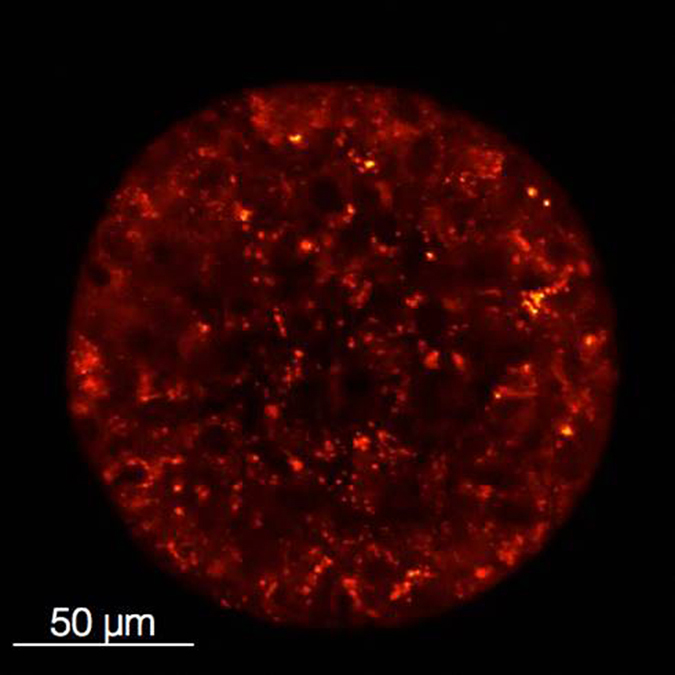
Uptake of PLGA-EtNBS nanoparticles into ovarian cancer *in vitro* spheroids (OVCAR5). PLGA-EtNBS nanoparticles are distributed throughout all cells within *in vitro* tumour spheroids, indicating that the nanoparticles diffuse and are transported through multiple cell layers. The dimmer appearance of EtNBS fluorescence in the centre of the spheroid relative to the periphery is not reflective of the actual cellular uptake and is the result of light scattering within the solid cellular mass. The distribution of PLGA-EtNBS under confocal microscopy matches that observed under identical conditions for free EtNBS, where the photosensitizer was found to be taken up throughout the spheroid.

In order for PE nanoparticles to be effective within hypoxic cellular microenvironments, the particles themselves must also be capable of diffusing into these deep acidic tumour regions. While EtNBS was found to readily diffuse along pH gradients into hypoxic tumour environments, it was not known if PE nanoparticles would have the same ability. Three-dimensional OVCAR5 tumour nodules, grown for a period of 10 days, are known to contain both hypoxic and acidic cores^28^,^29^. 3D cultures were incubated with PE nanoparticles for 4.5 h, and then visualized using confocal microscopy. As can be seen in Fig. 6, EtNBS fluorescence can be observed in cells throughout the spheroid within punctate, perinuclear lysosomes. When continually illuminated with raster-scanned 635 nm laser light, EtNBS within 3D spheroid cells was observed to photobrighten, indicating the release of EtNBS from the nanoparticles (see Supplementary Video S2). Furthermore, similar to that observed in monolayer cultures, EtNBS photobrightening is accompanied by localization changes from punctate particles to a more cytoplasmic distribution. These results demonstrate that PLGA-EtNBS nanoparticles are able to diffuse throughout 3D spheroids, as free EtNBS does.

## Discussion

PLGA nanoparticle encapsulation of EtNBS is a successful strategy for overcoming dark toxicity, a critical issue currently limiting EtNBS and similar cationic photosensitizers. Moreover, this encapsulation approach did not result in a loss of function or efficacy, with nanoparticles retaining the same cellular delivery and photodynamic therapy efficiency via a light-mediated release mechanism. The significant reduction in dark toxicity is an important step in advancing EtNBS and its family of compounds^29^ towards pre-clinical and clinical application.

A particularly exciting finding was that PLGA-encapsulated EtNBS nanoparticles are potent in both normoxia and hypoxia. Many chemotherapeutics, including taxanes, anthracyclines, and platinum compounds, as well as ionizing radiation, experience reduced cytotoxic potential under low oxygenation, making hypoxia a significant challenge in modern cancer treatment. EtNBS, in contrast, has been found to have an appreciable advantage in this regard, as it has been recognized to retain tumour-killing effects even under severely hypoxic conditions^29^,^44^,^45^. As hypoxic tumour regions are thought to promote more aggressive phenotypes and perhaps harbour tumour initiating cells, EtNBS PDT represents a potential route to reducing local tumour recurrence and metastasis. Since PLGA nanoparticle encapsulation of EtNBS reduced dark toxicity, PLGA nanoparticle formulations may present a safer administration approach for the treatment of hypoxic tumour regions. It is worth noting that the strong fluorescence of EtNBS may even be leveraged in such applications *in vivo* to permit facile tracking of EtNBS distribution and lysosomal disruption throughout tumours.

A logical extension of this photosensitizer-PLGA encapsulation approach is the co-encapsulation of other photosensitizers or therapeutics to treat cells within normally unresponsive tumour microenvironments. While photodynamic release has been demonstrated with Type II, oxygen-consuming photosensitizers, such formulations would likely not be effective within hypoxic compartments. The efficacy of PLGA-EtNBS nanoparticles could pave the way for the encapsulation and release of drugs such as mitochondria-targeting or caspase-mediated cell death signalling pathway molecules, which have been shown to act synergistically with photosensitizers in previous PDT studies^46^,^47^. Extended PDR applications include the activation of multiple cargo complexes within the nanoparticle by different wavelengths of light at separate points in time for custom, highly-specific treatment plans for personalized medicine applications.

Also exciting is the highly extensible nature of PLGA nanoparticles; with further modifications to either their surface or cargo, these PLGA-EtNBS nanoparticles could target individual tumours or cell types within tumours via specific antibodies or aptamers. This is of particular interest in OvCa, where intraperitoneal PDT has been associated with bowel toxicity in animal studies. Targeting these nanoparticle platforms to specific cancer cell types and their associated stroma could significantly decrease collateral toxicity and may improve treatment outcomes^48^.

## Methods

### Monolayer cell culture

The OVCAR5 human ovarian cancer cell line (Fox Chase Cancer Institute) was used throughout all experiments. OVCAR5 cells were maintained in a complete RPMI 1640-glutamine containing medium supplemented with 10% heat inactivated foetal bovine serum (FBS, Gibco, Invitrogen) and 1% antibiotics (penicillin-streptomycin 5000 IU/mL, CellGrow, MediaTech). Cells were passaged regularly every 2–3 days with 0.05% trypsin (CellGrow, MediaTech). Trypsin was added to the cell cultures following a 20 min incubation period with calcium- and magnesium-free Dulbecco’s phosphate-buffered saline (DPBS, CellGrow, MediaTech).

### Three-dimensional cell culture

Three-dimensional spheroid cultures created from OVCAR5 cells were prepared following previously published protocols in 35 mm glass-bottom plates (P35G-0-14, MatTek)^29^.

### Synthesis of EtNBS and EtNBS-NH2

EtNBS and the amine-terminated 2-amino-N-(9-(diethylamino)-5H-benzo[a]phenothiazin-5-ylidene)ethanaminium chloride (EtNBS-NH_2_) were synthesized following previously described procedures^49^. Prior studies have found the amine-terminated EtNBS derivative to have identical photochemical properties to the parent molecule^29^.

### Preparation of PLGA-encapsulated EtNBS nanoparticles (PE)

To form PLGA nanoparticles that physically encapsulate EtNBS, PLGA (25 mg, MW 7,000–17,000, Sigma-Aldrich), *N,N*-dimethylformamide (DMF, 1 mL, Acros Organics) and EtNBS (1 mg) in methanol (250 μL, Acros Organics) were mixed in a round bottom flask with a stir bar. The solution was rigorously mixed until homogeneous. This EtNBS solution was then added drop-wise into a stirred solution of bovine serum albumin (BSA, 100 mg, Sigma-Aldrich) in water (10 mL) to create the PLGA nanoparticles by nanoprecipitation^50^. The resulting solution was then immediately sonicated for 1 min. DMF and methanol were removed from the solution by rotary evaporation. The nanoparticle solution was centrifuged at 4,000 rpm for 15 min at 25 °C on a Sorvall RT 6000 to pellet and remove any large PLGA aggregates. The resulting supernatant was then processed through three rounds of serial centrifugation. At each round, the supernatant was transferred to Beckman Coulter Optiseal ultracentrifuge tubes and centrifuged at 12,000 rpm for 10 min at 25 °C on Beckman Coulter Optimal L-90 K ultracentrifuge. The pellet was retained upon each round of centrifugation, re-dissolved in water (1 mL), and stored at 4 °C. To form unloaded PLGA nanoparticles, the same procedure was followed with the EtNBS solution replaced with an equivalent volume of methanol. Nanoparticle size and zeta potential were measured via dynamic light scattering (Nano ZS, Malvern).

Initially, it was found that residual free EtNBS existed in solution immediately following nanoprecipitation, even after multiple rounds of washing on standard cell culture centrifuges. To remove this excess free EtNBS, ultracentrifugation was tested until the optimal *g* value to reversibly pellet the nanoparticles was determined. Dynamic light scattering was used to confirm nanoparticle size after each ultracentrifugation step. Ultimately, this resulted in a protocol that allows for the isolation of nanoparticles of consistent size across synthetic batches without the presence of free EtNBS.

### Preparation of chemically-conjugated PLGA-EtNBS nanoparticles (CC)

*N*-hydroxysuccinimide-terminated PLGA (PLGA-NHS) was synthesized from the same PLGA stock as above following a previously published protocol^44^. To conjugate EtNBS-NH_2_ to PLGA-NHS, PLGA-NHS (43.5 mg), EtNBS-NH_2_ (3.25 mg) and *N,N*-diisopropylethylamine (18 mg) were added to DMF (3 mL) in a flask with a stir bar and stirred for 2 h. The resulting solution was dried by rotary evaporation. The dry crude product EtNBS-NH-PLGA was washed with water (3 × 1 mL) until the extracts were no longer blue. To make the chemically-conjugated EtNBS-NH-PLGA nanoparticles, 8 mg of EtNBS-NH-PLGA was added to 417 μL of DMF. This mixture was then added dropwise into a stirring solution of BSA (3.3 mL, 10 mg/ml in H_2_O) and then sonicated for 1 min before removing DMF by rotary evaporation. The nanoparticle solution was centrifuged at 4,000 rpm for 15 min at 25 °C on a Sorvall RT 6000. The resulting supernatant was centrifuged under these conditions three times. Ultimately, the supernatant was transferred to Beckman Coulter Optiseal ultracentrifuge tubes and centrifuged at 12,000 rpm for 10 min at 25 °C on a Beckman Coulter Optimal L-90K ultracentrifuge. The pellet was retained, dissolved in water (1 mL) and stored at 4 °C. Nanoparticle size and zeta potential were measured via dynamic light scattering (Nano ZS, Malvern).

### Nanoparticle loading

Once loaded into nanoparticles, the proximity between individual EtNBS molecules is such that the molecules quench each other, likely though a ground state quenching mechanism^43^. This prevents straightforward determination of loaded EtNBS levels and requires the nanoparticles to be broken up in order to extract EtNBS for quantification. EtNBS loading within PLGA nanoparticles was measured by adding 10 μL of nanoparticle solution into a solution of 490 μL dichloromethane (DCM) and 500 μL acidified methanol followed by immediate homogenization with a probe sonicator until a clear, homogenous solution was obtained. The EtNBS concentration was then determined by diluting the obtained solution in acidified methanol and using a fluorometer (Eclipse, Cary) set for excitation at 630 nm and emission at 650–750 nm, with the total intensity measured by calculating the area-under-the-curve (AUC) of the emission band. Using known concentration standards, this AUC value was converted into EtNBS concentration.

### Cellular EtNBS uptake and extraction

EtNBS and structurally similar compounds have been previously reported to bind to plastic surfaces^40^. To prevent any loss of compound, cellular uptake of EtNBS was carried out in suspension throughout the experiments. Briefly, OVCAR5 cells were trypsinized and 1,000,000 cells were pelleted at 1,000 rpm for 5 min. The resulting cell pellet was reconstituted in 1 mL complete RPMI 1640 medium with either EtNBS or EtNBS-encapsulated PLGA nanoparticles with final concentrations of 0.5 μM, 1 μM, 2.5 μM, 5 μM, and 10 μM. Cell-compound suspensions were incubated at 37 °C with 5% CO_2_ and controlled humidity for 1.5 h. Suspensions were vortexed every 10 min to avoid EtNBS binding to the plastic tube walls. Following incubation, cells were centrifuged at 1,000 rpm for 5 min, cell pellets were washed twice with DPBS without Ca^2+^/Mg^2+^, and pellets were subsequently collected for cellular EtNBS uptake extraction. EtNBS was extracted by adding 500 μL of acidified methanol and 500 μL of DCM to the collected pellets, followed by homogenization with a probe sonicator. Cellular EtNBS intensity was integrated as described above using a fluorometer and determining the AUC. Experiments were conducted in triplicate.

### MTT monolayer cellular viability

OVCAR5 cellular response to therapy was measured using the MTT colorimetric assay following a published protocol^29^. The MTT assay is a standard assay performed in the evaluation of PDT in cell culture. It should be noted that the MTT assay reports viability via mitochondrial oxidoreductase enzyme activity, where tetrazole is reduced into insoluble purple formazan in living cells. Compromised and non-viable cells lose mitochondrial activity, which can be detected via the MTT assay. When carefully tested and standardized across cell lines and experimental replicates, the MTT assay is a reliable method for reporting viable cell counts that correlates well with clonogenic assays^51^.

### Dark toxicity measurement

OVCAR5 cells were plated in 24 well dishes at a density of 50,000 cells per well with complete RPM 1640 medium. 24 h later, the cells were incubated for 1.5 h at 37 °C, 5% CO_2_, with concentrations of 0.5 μM, 1 μM, 2.5 μM, 5 μM, and 10 μM of either EtNBS alone or the equivalent concentration of EtNBS in PLGA-EtNBS nanoparticles. Next, the solutions in each well were replaced with fresh complete RPMI 1640 medium for an additional 24 h incubation in the dark. At the end of the incubation period, cell viability was measured by the MTT assay. Experiments were conducted in triplicate.

### Monolayer PDT efficacy

OVCAR5 cells were seeded in 24-well plates at 50,000 cells per well 24 h prior to the experiment. Cells were loaded with either EtNBS or EtNBS-encapsulated PLGA nanoparticles with EtNBS concentrations ranging from 0.5 μM to 10 μM and incubated for 1.5 h at 37 °C with 5% CO_2_ under controlled humidity. Prior to light exposure, each well was replaced with fresh complete medium. A fluence of 100 mW/cm^2^ from a 660 nm diode was used to irradiate cells throughout PDT experiments (M660L2, ThorLabs) for 10, 50, or 100 seconds to obtain 1 J/cm^2^, 5 J/cm^2^, or 10 J/cm^2^ light doses, respectively. The 24-well plate was incubated for an additional 24 h at 37 °C prior to the evaluation of cell response by the MTT-mediated cell viability assay.

### Establishing and monitoring hypoxic conditions

An oxygen partial pressure of less than 10 mmHg pO_2_ was considered to be a hypoxic condition^52^. To achieve this level of oxygenation, a gas-tight hypoxia chamber (Stem Cell Technologies) was used to maintain a partial pressure of ≤3 mmHg pO_2_ in the extracellular environment throughout the experiment. The oxygen partial pressure was continuously monitored by an oxygen sensor probe (UniSense, OxyMeter) placed within the chamber. 24 h after plating, dishes were placed inside the chamber and purged with 5% CO_2_ balance nitrogen gas for 10 min until the pO_2_ within the chamber reached a stable partial pressure of ≤3 mmHg. Next, the hypoxic chamber was sealed and placed into a 37 °C incubator for 6 h, and subsequently re-purged for 10 min with 5% CO_2_ balance nitrogen every hour until the final time point. The oxygen levels within the chamber were carefully monitored throughout this incubation period to ensure that the oxygen partial pressure within the chamber was maintained at ≤3 mmHg pO_2_. For experiments under hypoxic conditions, cells were first placed under hypoxia for 6 h, at which point the cells were loaded with either EtNBS or EtNBS-encapsulated PLGA nanoparticles with varied concentrations in complete RPMI 1640 medium bubbled by 5% CO_2_ balance nitrogen. After loading cells with the compounds, the cell plate was placed back into the hypoxic chamber and purged again to a pO_2_ partial pressure ≤3 mmHg. Cells were then incubated with either EtNBS or PLGA-EtNBS nanoparticles under hypoxic conditions inside the hypoxic chamber for 1.5 h. For the hypoxic dark toxicity study, following EtNBS/PLGA-EtNBS incubation, the cell medium was replaced with fresh medium and cells were incubated at 37 °C with 5% CO_2_ and controlled humidity for an additional 24 h. For the hypoxic PDT experiment, cells were first incubated for 1.5 h under hypoxic conditions with either EtNBS or PLGA-EtNBS. Next, the cell medium was replaced with fresh 5% CO_2_ balance nitrogen-purged complete medium prior to subsequent purging within the chamber with 5% CO_2_ balance nitrogen. Finally, while still in a hypoxia chamber, the cells were irradiated under 5% CO_2_ balance nitrogen gas atmosphere with light doses of 1 J/cm^2^, 5 J/cm^2^, or 10 J/cm^2^, as described above.

### Cellular localization

To observe cellular localization of the nanoparticles, OVCAR5 cells were trypsinized and plated in 35 mm dishes at 200,000 cells per dish 24 h prior to loading with nanoparticles. Cells were incubated with nanoparticles for 1.5 h, after which the cell medium was replaced with fresh complete medium. Lysotracker green dye was used to confirm lysosomal nanoparticle localization within the cell. For Lysotracker green labelling, OVCAR5 cells were loaded with 500 nM Lysotracker green DND-26 (excitation/emission maxima ~504/511 nm, Molecular Probes) for 1 h, and then replaced with complete fresh medium before imaging with confocal microscopy (Olympus FV1000).

### Spheroid incubation

OVCAR5 spheroids were grown for 10 days. At this time, they were then incubated with 500 nM EtNBS/PLGA-EtNBS in complete medium for 4.5 h. The cell medium was then replaced with fresh complete medium and the spheroids imaged on a confocal microscope.

### Confocal microscopy

All imaging experiments were carried out on an Olympus FV1000 confocal fluorescence microscope. Cellular images were acquired using either an Olympus UPlanSApo 20 × 0.75NA or an Olympus UPlanSApo 60 × 1.2NA objective. Spheroid images were acquired using an Olympus LUMPFl 40 × 0.8NA objective. EtNBS was excited using 635 nm light, with its emission collected between 650 and 750 nm. For long-term time-lapse experiments, samples were imaged on a heated incubator stage (Tokai-Hit) with 5% CO_2_, balance air gas.

### Statistical analysis

The JMP 9 software package (SAS) was used to perform statistical analysis. The Student’s *t*-test was used to analyse statistical significance. p < 0.05 was considered statistically significant.

## Additional Information

**How to cite this article**: Hung, H. *et al*. PLGA nanoparticle encapsulation reduces toxicity while retaining the therapeutic efficacy of EtNBS-PDT *in vitro*. *Sci. Rep*. **6**, 33234; doi: 10.1038/srep33234 (2016).

## Supplementary Material

Supplementary Information

Supplementary Video S1

Supplementary Video S2

## Figures and Tables

**Table 1:** Sizes and zeta potentials of PLGA nanoparticles.

	Nanoparticle diameter (nm)	Zeta potential (mV)
PLGA nanoparticles alone (PA)	138	−39.9
Physically encapsulated PLGA-EtNBS nanoparticles (PE)	167	−33.7
Chemically conjugated PLGA-EtNBS nanoparticles (CC)	212	−40.8

## References

[b1] Foster R., Buckanovich R. J. & Rueda B. R. Ovarian cancer stem cells: working towards the root of stemness. Cancer letters 338, 147–157, doi: 10.1016/j.canlet.2012.10.023 (2013). 23138176

[b2] Dong Y. . Paclitaxel resistance and multicellular spheroid formation are induced by kallikrein-related peptidase 4 in serous ovarian cancer cells in an ascites mimicking microenvironment. PloS one 8, e57056, doi: 10.1371/journal.pone.0057056 (2013). 23451143 PMC3581584

[b3] Agarwal R. & Kaye S. B. Ovarian cancer: strategies for overcoming resistance to chemotherapy. Nature reviews. Cancer 3, 502–516, doi: 10.1038/nrc1123 (2003). 12835670

[b4] Murakami A. . Hypoxia increases gefitinib-resistant lung cancer stem cells through the activation of insulin-like growth factor 1 receptor. PloS one 9, e86459, doi: 10.1371/journal.pone.0086459 (2014). 24489728 PMC3904884

[b5] De Milito A. & Fais S. Tumor acidity, chemoresistance and proton pump inhibitors. Future oncology 1, 779–786, doi: 10.2217/14796694.1.6.779 (2005). 16556057

[b6] Calorini L., Peppicelli S. & Bianchini F. Extracellular acidity as favouring factor of tumor progression and metastatic dissemination. Experimental oncology 34, 79–84 (2012). 23013757

[b7] McCarty M. F. & Whitaker J. Manipulating tumor acidification as a cancer treatment strategy. Alternative medicine review : a journal of clinical therapeutic 15, 264–272 (2010). 21155627

[b8] Lin S. C., Liao W. L., Lee J. C. & Tsai S. J. Hypoxia-regulated gene network in drug resistance and cancer progression. Experimental biology and medicine 239, 779–792, doi: 10.1177/1535370214532755 (2014). 24812122

[b9] Warfel N. A. & El-Deiry W. S. HIF-1 signaling in drug resistance to chemotherapy. Current medicinal chemistry 21, 3021–3028 (2014). 24735366 10.2174/0929867321666140414101056

[b10] Peppicelli S., Bianchini F. & Calorini L. Extracellular acidity, a “reappreciated” trait of tumor environment driving malignancy: perspectives in diagnosis and therapy. Cancer metastasis reviews 33, 823–832, doi: 10.1007/s10555-014-9506-4 (2014). 24984804

[b11] Ramanathan B. . Resistance to paclitaxel is proportional to cellular total antioxidant capacity. Cancer research 65, 8455–8460, doi: 10.1158/0008-5472.CAN-05-1162 (2005). 16166325

[b12] Mimeault M. & Batra S. K. Hypoxia-inducing factors as master regulators of stemness properties and altered metabolism of cancer- and metastasis-initiating cells. Journal of cellular and molecular medicine 17, 30–54, doi: 10.1111/jcmm.12004 (2013). 23301832 PMC3560853

[b13] Nelke K. H., Pawlak W., Leszczyszyn J. & Gerber H. Photodynamic therapy in head and neck cancer. Postepy higieny i medycyny doswiadczalnej 68, 119–128, doi: 10.5604/17322693.1088044 (2014). 24491903

[b14] Allison R. R. & Moghissi K. Photodynamic Therapy (PDT): PDT Mechanisms. Clinical endoscopy 46, 24–29, doi: 10.5946/ce.2013.46.1.24 (2013). 23422955 PMC3572346

[b15] Moghissi K. Photodynamic therapy for lung cancer 30 years on. Photodiagnosis and photodynamic therapy 10, 95, doi: 10.1016/j.pdpdt.2013.05.003 (2013). 23769273

[b16] Shirasu N., Nam S. O. & Kuroki M. Tumor-targeted photodynamic therapy. Anticancer research 33, 2823–2831 (2013). 23780966

[b17] Yamamoto M. . Improvement of the efficacy of 5-aminolevulinic acid-mediated photodynamic treatment in human oral squamous cell carcinoma HSC-4. Acta medica Okayama 67, 153–164 (2013). 23804138 10.18926/AMO/50408

[b18] Hasan T., Ortel B., Moor A. C. & Pogue B. W. Photodynamic therapy of cancer. Cancer medicine 7, 537–548 (2003).

[b19] Kato H. Photodynamic therapy for lung cancer—a review of 19 years’ experience. Journal of Photochemistry and Photobiology B: Biology 42, 96–99 (1998). 10.1016/s1011-1344(97)00128-0 9540215

[b20] Friedberg J. S. . Radical pleurectomy and intraoperative photodynamic therapy for malignant pleural mesothelioma. The Annals of thoracic surgery 93, 1658–1667 (2012). 22541196 10.1016/j.athoracsur.2012.02.009 PMC4394024

[b21] Zhu T. C., Finlay J. C. & Hahn S. M. Determination of the distribution of light, optical properties, drug concentration, and tissue oxygenation *in-vivo* in human prostate during motexafin lutetium-mediated photodynamic therapy. Journal of Photochemistry and Photobiology B: Biology 79, 231–241 (2005). 10.1016/j.jphotobiol.2004.09.013 PMC4470428 15896650

[b22] Bown S. . Photodynamic therapy for cancer of the pancreas. Gut 50, 549–557 (2002). 11889078 10.1136/gut.50.4.549 PMC1773165

[b23] Biel M. Advances in photodynamic therapy for the treatment of head and neck cancers. Lasers in surgery and medicine 38, 349–355 (2006). 16788923 10.1002/lsm.20368

[b24] Kostron H., Obwegeser A. & Jakober R. Photodynamic therapy in neurosurgery: a review. Journal of Photochemistry and Photobiology B: Biology 36, 157–168 (1996). 10.1016/s1011-1344(96)07364-2 9002253

[b25] del Carmen M. G. . Synergism of epidermal growth factor receptor-targeted immunotherapy with photodynamic treatment of ovarian cancer *in vivo*. Journal of the National Cancer Institute 97, 1516–1524, doi: 10.1093/jnci/dji314 (2005). 16234565

[b26] Arenas Y. . Photodynamic inactivation of Staphylococcus aureus and methicillin-resistant Staphylococcus aureus with Ru(II)-based type I/type II photosensitizers. Photodiagnosis and photodynamic therapy 10, 615–625, doi: 10.1016/j.pdpdt.2013.07.001 (2013). 24284119

[b27] Usuda J. . Photodynamic therapy (PDT) for lung cancers. Journal of Thoracic Oncology 1, 489–493 (2006). 17409904

[b28] Evans C. L. . Killing hypoxic cell populations in a 3D tumor model with EtNBS-PDT. PloS one 6, e23434, doi: 10.1371/journal.pone.0023434 (2011). 21876751 PMC3158086

[b29] Klein O. J., Bhayana B., Park Y. J. & Evans C. L. *In vitro* optimization of EtNBS-PDT against hypoxic tumor environments with a tiered, high-content, 3D model optical screening platform. Molecular pharmaceutics 9, 3171–3182, doi: 10.1021/mp300262x (2012). 22946843 PMC3538815

[b30] Georgakoudi I. & Foster T. H. Effects of the subcellular redistribution of two nile blue derivatives on photodynamic oxygen consumption. Photochemistry and photobiology 68, 115–122 (1998). 9679457

[b31] Frimberger A. E., Moore A. S., Cincotta L., Cotter S. M. & Foley J. W. Photodynamic therapy of naturally occurring tumors in animals using a novel benzophenothiazine photosensitizer. Clinical cancer research: an official journal of the American Association for Cancer Research 4, 2207–2218 (1998). 9748141

[b32] O’Riordan K., Akilov O. E., Chang S. K., Foley J. W. & Hasan T. Real-time fluorescence monitoring of phenothiazinium photosensitizers and their anti-mycobacterial photodynamic activity against Mycobacterium bovis BCG in *in vitro* and *in vivo* models of localized infection. Photochemical & photobiological sciences: Official journal of the European Photochemistry Association and the European Society for Photobiology 6, 1117–1123, doi: 10.1039/b707962a (2007). 17914486

[b33] McCall R. L. & Sirianni R. W. PLGA nanoparticles formed by single- or double-emulsion with vitamin E-TPGS. Journal of visualized experiments: JoVE, 51015, doi: 10.3791/51015 (2013). PMC4106449 24429733

[b34] Hu K. . Hyaluronic acid functional amphipathic and redox-responsive polymer particles for the co-delivery of doxorubicin and cyclopamine to eradicate breast cancer cells and cancer stem cells. Nanoscale 7, 8607–8618, doi: 10.1039/c5nr01084e (2015). 25898852

[b35] Sendil-Keskin D., Altunay H., Wise D. L. & Hasirci V. *In vivo* pain relief effectiveness of an analgesic-anesthetic carrying biodegradable controlled release rod systems. Journal of biomaterials science. Polymer edition 14, 497–514 (2003). 12901434 10.1163/15685620360674218

[b36] Bedouet L. . Synthesis of hydrophilic intra-articular microspheres conjugated to ibuprofen and evaluation of anti-inflammatory activity on articular explants. International journal of pharmaceutics 459, 51–61, doi: 10.1016/j.ijpharm.2013.11.004 (2014). 24231051

[b37] Yallapu M. M., Gupta B. K., Jaggi M. & Chauhan S. C. Fabrication of curcumin encapsulated PLGA nanoparticles for improved therapeutic effects in metastatic cancer cells. Journal of colloid and interface science 351, 19–29, doi: 10.1016/j.jcis.2010.05.022 (2010). 20627257

[b38] El-Gogary R. I. . Polyethylene glycol conjugated polymeric nanocapsules for targeted delivery of quercetin to folate-expressing cancer cells *in vitro* and *in vivo*. ACS nano 8, 1384–1401, doi: 10.1021/nn405155b (2014). 24397686

[b39] Luzio J. P., Pryor P. R. & Bright N. A. Lysosomes: fusion and function. Nature reviews. Molecular cell biology 8, 622–632, doi: 10.1038/nrm2217 (2007). 17637737

[b40] Cincotta L., Foley J. W. & Cincotta A. H. Phototoxicity, redox behavior, and pharmacokinetics of benzophenoxazine analogues in EMT-6 murine sarcoma cells. Cancer research 53, 2571–2580 (1993). 8495421

[b41] Georgakoudi I. & Foster T. H. Effects of the subcellular redistribution of two nile blue derivatives on photodynamic oxygen consumption. Photochemistry and photobiology 68, 115–122 (1998). 9679457

[b42] Wang C., Cheng L. & Liu Z. Upconversion nanoparticles for photodynamic therapy and other cancer therapeutics. Theranostics 3, 317–330, doi: 10.7150/thno.5284 (2013). 23650479 PMC3645058

[b43] Lovell J. F., Liu T. W., Chen J. & Zheng G. Activatable photosensitizers for imaging and therapy. Chemical reviews 110, 2839–2857, doi: 10.1021/cr900236h (2010). 20104890

[b44] Karve S. . Formulation of diblock polymeric nanoparticles through nanoprecipitation technique. Journal of visualized experiments: JoVE, doi: 10.3791/3398 (2011). PMC3230208 21968609

[b45] Jung Y., Nichols A. J., Klein O. J., Roussakis E. & Evans C. L. Label-Free, Longitudinal Visualization of PDT Response *In Vitro* with Optical Coherence Tomography. Israel journal of chemistry 52, 728–744, doi: 10.1002/ijch.201200009 (2012). 23316088 PMC3538822

[b46] Hung H. I., Schwartz J. M., Maldonado E. N., Lemasters J. J. & Nieminen A. L. Mitoferrin-2-dependent mitochondrial iron uptake sensitizes human head and neck squamous carcinoma cells to photodynamic therapy. The Journal of biological chemistry 288, 677–686, doi: 10.1074/jbc.M112.422667 (2013). 23135267 PMC3537066

[b47] Kessel D. & Reiners J. J. Jr. Enhanced efficacy of photodynamic therapy via a sequential targeting protocol. Photochemistry and photobiology 90, 889–895, doi: 10.1111/php.12270 (2014). 24617972 PMC4082464

[b48] Spring B. Q. . A photoactivable multi-inhibitor nanoliposome for tumour control and simultaneous inhibition of treatment escape pathways. Nature nanotechnology 11, 378–387, doi: 10.1038/nnano.2015.311 (2016). PMC4821671 26780659

[b49] Verma S. . Antimicrobial photodynamic efficacy of side-chain functionalized benzo[a]phenothiazinium dyes. Photochemistry and photobiology 85, 111–118, doi: 10.1111/j.1751-1097.2008.00403.x (2009). 18657053

[b50] Betancourt T., Brown B. & Brannon-Peppas L. Doxorubicin-loaded PLGA nanoparticles by nanoprecipitation: preparation, characterization and *in vitro* evaluation. Nanomedicine 2, 219–232, doi: 10.2217/17435889.2.2.219 (2007). 17716122

[b51] Kawada K. . Comparison of chemosensitivity tests: clonogenic assay versus MTT assay. Acta medica Okayama 56, 129–134 (2002). 12108583 10.18926/AMO/31714

[b52] Hockel M. & Vaupel P. Tumor hypoxia: definitions and current clinical, biologic, and molecular aspects. Journal of the National Cancer Institute 93, 266–276 (2001). 11181773 10.1093/jnci/93.4.266

