# Supplementary material for: PLGA nanoparticle encapsulation reduces toxicity while retaining the therapeutic efficacy of EtNBS-PDT in vitro
Source: Sci Rep. 2016 Sep 30;6:33234. doi: 10.1038/srep33234 (PMC5043181; doi:10.1038/srep33234)
Supplement: Supplementary Information [file srep33234-s1.pdf]

PLGA nanoparticle encapsulation reduces toxicity while retaining the therapeutic efficacy of EtNBS-PDT *in vitro*

Hsin-I Hung[1], Oliver J. Klein[1], Sam W. Peterson[1], Sarah R. Rokosh[1], Sam Osseiran[1,2], Conor L. Evans[1]*

[1]Wellman Center for Photomedicine, Harvard Medical School, Massachusetts General Hospital, 149 13th Street, Charlestown, Massachusetts 02129, United States.
[2]Harvard-MIT Division of Health Sciences and Technology, 77 Massachusetts Avenue E25-519, Cambridge, Massachusetts 02139, United States.

*Corresponding Author:

Conor L. Evans, PhD
CNY149-3210
13th Street
Massachusetts General Hospital
Charlestown, MA 02129
Phone: 617-726-1089
Fax: 617-726-4453
evans.conor@mgh.harvard.edu

Supplementary Video S1: Photobrightening of PLGA-EtNBS nanoparticles administered to ovarian cancer cell monolayers *in vitro*. PLGA-EtNBS nanoparticles are localized to the cells' lysosomes. Upon photoexcitation with focused 635 nm laser light, fluorescence centered at 670 nm is generated and collected over time. Over the 3.5-minute time course (shown at 21 frames per second; each frame was collected with an acquisition time of 3.33 seconds), the fluorescence distribution changes from initially punctate and localized to the lysosomes, to bright and diffuse across the entire cytosol.

Supplementary Video S2: Photobrightening of PLGA-EtNBS nanoparticles administered to ovarian cancer spheroids *in vitro*. EtNBS is photoexcited with focused 635 nm laser light, producing fluorescence centered at 670 nm. The brightness of the fluorescence signal can be seen to increase over time following continuous scanning of 635 nm laser light. Note that the subcellular localization of EtNBS changes throughout photobrightening, from initially being confined to lysosomes to being more evenly distributed throughout each cell.
